# The Inhibitory Effect of the Deregulation Short-Sale on Private Equity Placement

**DOI:** 10.3389/fpsyg.2022.888805

**Published:** 2022-07-12

**Authors:** Panna Wang, Chun Su, Botao Xu

**Affiliations:** Accounting School, Chongqing University of Technology, Chongqing, China

**Keywords:** deregulation on short-sale constraint, company private placement, information environment, second type agency problem, corporate sustainability

## Abstract

In countries with a high degree of equity concentration, such as China, the second type of agency problem is serious in which major shareholders of listed companies encroach on the interests of small and medium shareholders through private placement. As a financial transaction system, the short-selling mechanism has the function of external corporate governance. Therefore, this paper examines the impact of relaxing short-selling controls on companies’ private placements. Based on the quasi-natural experiment of China’s gradual relaxation of short-selling controls since 2010, a double-difference model (DID) test is constructed to find that relaxing short-selling controls reduces companies’ private placement tendency and amount, and this effect is mainly reflected in samples with a higher degree of equity checks and balances. Further analysis found that when the size of the company’s financing constraints is different, the impact of relaxing short-selling control on the company’s private placement tendency and amount is different; when the chairman and the general manager have two positions, relaxing short-selling control reduces the company’s private placement options, but it has a negative impact on the company’s private placement. There is no difference in the impact of the company’s private placement amount. It can be excluded that the relaxation of short-selling control suppresses the company’s placement through financing constraints and corporate governance mechanisms. Compared to a good information environment, when the company’s information environment is poor, relaxing short-selling controls reduces the company’s private placement tendency and amount. Significantly, it shows that the relaxation of short-selling control policy and supervision inhibits the company’s private placement through the information environment mechanism, and promotes the sustainable development of enterprises. It not only improves the literature in the field of short-selling mechanism affecting corporate behavior, but it also enriches the research on the transfer of benefits of private placement of companies.

## Introduction

China’s A-share market raised 920 billion yuan through additional offerings in 2015, about six times that of IPOs. It can be seen that private placement is one of the most important ways of equity refinancing of Chinese listed companies, and it is also an important financial decision-making behavior of the company. Existing literature confirms that there is a behavior of “transferring benefits” to major shareholders in the process of private placement of companies, especially in countries with poor investor protection (Chen and Liu, 2014; Rennekamp et al., 2020). China’s listed companies have a high degree of equity concentration, the legal protection of investors in the capital market is not perfect, and the agency problem between the controlling major shareholder and external small and medium shareholders is serious. Existing studies have found that major shareholders realize the benefit transfer of directional additional issuance by lowering the additional issuance price, injecting inferior assets, wealth transfer, earnings management and high-proportion discounts (Wang et al., 2010; Zhang, 2010; Zhang and Li, 2010). Investor protection is of great significance to promoting the development of the financial market, so it is of great practical significance to discuss how to deal with the encroachment of interests such as private placement.

As an innovative financial transaction system, the short-selling mechanism not only affects the pricing efficiency of the stock market, but it also plays a role in corporate governance (Karpoff and Lou, 2010; Rennekamp et al., 2020; Liu et al., 2021). According to the design of the short-selling mechanism trading system, investors are encouraged to tap the company’s “bad news” to make investment profits, and short-selling investors have a strong incentive to short-sell the stocks of companies with misconduct such as interest encroachment to obtain investment returns (Massa et al., 2015; Stephana et al., 2021). Therefore, in order to avoid losses caused by short selling, the major shareholders or management of the short-selling company will restrain their own misconduct. Existing scholars have studied the external governance effect of short-selling mechanism on listed companies from different perspectives. These governance paths include restraining the company’s major shareholders and management’s interests, such as embezzling company cash (Hou et al., 2016), excessive investment (Chang et al., 2015), corporate violations (Meng et al., 2019), m&a activity (Wei et al., 2020), related-party transactions (Jiang et al., 2020; Stephana et al., 2021), and other misconduct. Shleifer and Vishny (1997) proposed the second type of surrogate problem, and then LLSV et al. studied these problems from various perspectives. Tian (2001) and Tenev et al. (2002) found that the second type of agency problems in emerging markets such as China are more prominent, such as the acquisition of funds by major shareholders and private placement, the governance of the second type of agency problem in emerging markets such as China has become an academic hotspot. Existing scholars have studied the external governance effect of short-selling mechanism on listed companies from different perspectives. At present, there is no research to empirically test the governance effect of the short-selling mechanism on the company’s private placement. Based on this, this paper uses an empirical method to investigate whether the relaxation of short-selling control has an inhibitory effect on the company’s private placement, and further explore which shareholding structure sample is mainly reflected in the relaxation of short-selling control on the company’s private placement and the difference in the above-mentioned effects when the company’s information environment is different.

As an emerging market country with weak investor protection, China’s major controlling shareholder transfers interests through private placement and encroaches on the interests of small and medium shareholders, which seriously hinders the development of the financial market. Therefore, the research question of this paper is whether the short-selling mechanism can play a governance role in inhibiting the company’s private placement. Taking the A-share listed companies in Shanghai and Shenzhen stock exchanges from 2007 to 2015 as a sample, a double-difference model is constructed to test and find that the relaxation of short-selling control reduces the company’s private placement tendency and amount, and this effect is mainly reflected in the samples with a high degree of equity checks and balances. Further analysis found that in the samples with poor information environment, the relaxation of short-selling control prompted the company to reduce the behavior of privately offering benefits, which proved that the information environment was the main mechanism that affected the relaxation of short-selling control and reduced the company’s additional issuance interest encroachment.

The contributions of this paper are mainly reflected in the following three aspects: First, it improves the literature on the impact of short-selling mechanism on corporate behavior. Existing literature confirms the impact of short-selling mechanisms on corporate investment decisions, earnings management, and innovation. This paper finds that the short-selling mechanism reduces the company’s private placement behavior, enriches the short-selling mechanism policy supervision system, and affects the literature on corporate agency behavior. Second, it expands the research on the external governance of the company’s targeted additional issuance of benefits. Most of the existing researches on the private placement of companies provide evidence of the transfer of interests of major shareholders. This paper discusses the governance of the agency problem of private placement. Combined with the quasi-natural experimental opportunity of China’s launch of the short-selling mechanism, it proves that the short-selling mechanism can play a role. The role of external governance can reduce the second-type agency problem behavior of the company’s major shareholders who transfer the benefits of private placement. Third, it enriches the research on sustainable development of enterprises. This paper finds that the short-selling mechanism plays an external governance role, inhibits the company’s private placement of benefits, and obtains the capital market policy supervision system to promote the sustainable development of enterprises.

## Literature Review and Research Hypotheses

### Company Private Placement Related Literature

Private placement refers to the non-public issuance of shares by listed companies to specific objects. This financing method is flexible. It is one of the most important methods for equity refinancing of listed companies in China, and it is also an important financial decision-making behavior of the company. Due to the strict restrictions imposed by the competent government departments on the objects of additional issuance and the circulation of shares, the additional issuance objects are vulnerable to market risks and losses caused by stock fluctuations during the long period of restricted stock sales. Therefore, additional issuance objects such as major shareholders have the motive to compensate market risks by seeking additional benefits. Existing literature has confirmed that the company’s major shareholders carry out the phenomenon of “interest transmission” through private placement, especially in countries with poor investor protection, the above-mentioned “interest transmission” phenomenon is more serious (Chen and Liu, 2014; Rennekamp et al., 2020). The so-called transfer of interests refers to the transfer of the interests of the company by the major shareholders of the listed company into personal interests, which constitutes the act of encroaching on the interests of small and medium shareholders or outsiders. The legal protection of investors in China’s capital market is not perfect, the equity concentration of listed companies is high, and the agency problem between the controlling shareholder and external small and medium shareholders is serious. Regarding the transfer of interests by the company’s major shareholders through private placement, scholars have found that it is mainly reflected in the following aspects: (1) Before the private placement, the major shareholders use timing and trading suspension to control and lock in a lower price for benefit transfer. Major shareholders suppress the stock price before the benchmark date by suspending trading for a long time before private placement, and conduct additional issuance to themselves or other related parties at a lower price in order to transfer benefits (Wu et al., 2011). (2) Realize the benefit transfer of private placement by injecting inferior assets, wealth transfer and earnings management (Zhang, 2010; Zhang and Li, 2010). (3) Use a high percentage of discount to transfer benefits, confirming that the additional issuance price is closely related to the identity of the additional issuance object and the shareholding ratio of major shareholders, so as to realize wealth transfer through related transactions after private issuance (Zhang and Li, 2008; Li and Jian, 2017; Ni et al., 2020).

### Short-Selling Mechanism Related Literature

As a financial transaction system, the short-selling mechanism gives investors the right to profit from the company’s “bad news” investment. Existing literature has confirmed that short-selling mechanism plays the information effect in the market (Li and Liu, 2021), also as an external governance mechanism, inhibits corporate earnings management (Liu et al., 2021), which is reflected in inhibiting over-investment (Chang et al., 2015), adjusting investment decisions (Jin et al., 2015), promoting corporate innovation (Quan and Yin, 2017), reducing financing behavior (Gu and Zhou, 2017), improving corporate investment efficiency (Wang and Wang, 2018), and improve the quality of corporate environmental information disclosure, increase in hard disclosures especially (Xie et al., 2021). Faced with the increased risk of stock price downside caused by the short-selling mechanism, the company’s management may reduce the accuracy of performance forecasts (Li and Zhang, 2015), disclose the company’s “bad news” in a timely manner (Clinch et al., 2016), shorten the bond issuance period to transmit signals to improve the market information environment (Wang et al., 2020), influence investor behavior, and the severity of fully impounding negative information in the short run (Huang et al., 2021; Li and Liu, 2021). China’s securities market has launched a short-selling mechanism in 2010, and there is abundant evidence on the governance effect of deregulation of short-selling on Chinese listed companies. For example, Chen and Liu (2014) found that based on the quasi-natural experiment of China’s short-selling mechanism, the accrued earnings management and real earnings management of short-selling target companies were significantly lower, which confirmed that the short-selling mechanism played an external governance role to restrain the company’s earnings, and manage opportunistic behavior. Hou et al. (2016) showed that the relaxation of short-selling control restricts the misappropriation of major shareholders’ interests. If the loss from decline in stock price caused by short-selling is greater than the profit from misappropriation of shareholders’ interests, the possibility of cash assets being misappropriated is reduced, which increases the value of cash, and is mainly reflected in the private enterprises with a relatively high shareholding ratio of the largest shareholder.

### Research Hypothesis

The information environment of listed companies in emerging market countries such as China is relatively complex, the equity concentration is high, and the second type of agency problem is more serious in which large shareholders encroach on the interests of small and medium shareholders. Therefore, it is of great practical significance to discuss how to control the large shareholders encroaching on the interests of small and medium shareholders through private placement. Chang et al. (2015) and Wang and Wang (2018) all found that the short-selling mechanism, as a regulatory policy, has a role in corporate external governance. Within the company, although the major shareholders use their own information advantages and control rights to transfer interests through private placement, so that they can obtain substantial benefits, after the relaxation of short-selling control, if major shareholders continue to seek personal interests through private placement, they may be punished by investors for short-selling the company’s stock. If the company is short-sold and the stock price falls sharply, it will harm the interests of major shareholders, and take financial risk (Field, 2022). In particular, the China securities market is very speculative, and if the stock price falls sharply, it may cause the risk of a stock price crash. Liu et al. (2021) found that potential short-selling opportunities can effectively suppress earnings manipulation and improve earnings quality. Therefore, after the relaxation of short-selling control, major shareholders will weigh the gains and the corresponding losses caused by short-selling (Gu and Zhou, 2017). If the gain from the private placement is less than the loss from the stock price drop caused by being short-sold, the major shareholder will choose to reduce the frequency of the private placement and the amount of the private placement, that is, the relaxation of short-selling control has an inhibitory effect on the “transfer of benefits” by major shareholders through private placement. Based on the above analysis, this paper proposes hypothesis 1.

*H1*: Controlling other factors and relaxing short-selling controls will reduce the company’s tendency to issue private placements and reduce the number of private placements.

In the company’s internal equity structure, equity checks and balances represent the sharing of control rights among several major shareholders. Through internal restraint, no major shareholder can independently control the company’s decision-making, and the major shareholders can supervise each other. When the degree of equity checks and balances is high, the role of major shareholders in mutual supervision is stronger. In the scenario of deregulation of short-selling, major shareholders will face the risk of being short-sold by investors if they seek personal gain through private placement. At this time, the major shareholders will weigh the gains from the private placement and the loss of being short-sold. When the private benefit of the private placement is less than the loss of being short-sold, they will give up the self-interested behavior of the private placement. Based on the observation of Chinese listed companies, the relaxation of short-selling control restricts the encroachment of the interests of large shareholders on the hollowing out of small and medium shareholders, that is, the short-selling mechanism, as a financial transaction system, forms an External Oversight Mechanism for the second type of agency problem of the hollowing out behavior of large shareholders (Massa et al., 2015; Hou et al., 2016). Due to the reduced possibility of private placement of the target company that relaxes short-selling control, the market’s discount for private placement of the target company for which short-selling control is relaxed will be reduced compared to companies that have not relaxed short-selling control. Accordingly, hypothesis 2 is proposed here.

*H2*: High degree of equity checks and balances Compared with samples with low equity checks and balances, relaxing short-selling controls has a more significant effect on reducing the company's directional issuance tendency and amount.

## Research Design

### Study Sample Selection and Data Sources

All the company’s financial data and short-selling data in this article come from the Guotai’an (CSMAR) database, and the company’s private placement data comes from the Wind (WIND) database. The initial sample of the study is all A-share listed companies in Shanghai and Shenzhen stock markets. The financial and insurance industries are excluded from the sample screening process, and ST or ^*^ST handles samples and samples with missing variables. Since China launched the short-selling mechanism in March 2010, the number of short-selling targets has been expanded five times to 900 by 2014. In order to construct a double difference model for comparison, the sample period selected in this paper is from 2007 to 2015. The nonfinancial companies that did not enter the short-selling target among the A-share listed companies on the Shanghai and Shenzhen stock exchanges were taken as the control group. All samples of listed companies listed in the current year and after 2009 were excluded. For the main continuous variables, in order to eliminate the influence of extreme values, the adjustment Winsor was performed according to the 1% quantile. In order to ensure the robustness of the regression results, all regressions are subject to robustness Robust control.

### Model Building

The gradual relaxation of short-selling control in China’s capital market is a quasi-natural experiment of multiple shocks, in order to accurately measure the impact of the relaxation of short-selling control on the company’s additional issuance. According to the classic double-difference model, the short-selling time point (Post) item should be added to the model to participate in the regression, but in this quasi-natural scenario of China’s gradual relaxation of short-selling control, the short-selling time point (Post) and the short-selling time point are short-selling The list cross item (Post^*^List) has a serious multicollinearity problem, which makes it impossible to display the regression coefficient of the short-selling list cross item (Post^*^List) when the key observation variable is short-selling. After consulting relevant literature, this paper refers to Bertrand and Mullainathan (1999) and Chu and Fang (2016) proposed the double-difference model to construct a multi-time-point double-difference (DID) model, which controls other variables that may affect the company’s private placement. Since the dependent variable company’s propensity for additional issuance (SEO) is a dummy variable, the probit regression method is used to construct the model (1).


(1) $$\begin{array}{l} \mathrm{Pr}obit\left(SEO\right)=\partial_{0}+\partial_{1}List+\partial_{2}Post^{*}List+\partial_{3}Size+\partial_{4}Lev+\partial_{5}Tobinq\\ +\partial_{6}Roa+\partial_{7}Cfo+\partial_{8}Growth+\partial_{9}Dual+\partial_{10}Instu+\partial_{11}Big\\ +\partial_{12}Disp+\partial_{13}Soe+\partial_{14}\sum year+\partial_{15}\sum ind+\varepsilon \end{array}$$

In order to detect the impact of the relaxation of short-selling control on the company’s private placement amount, the dependent variable at this time is the company’s additional issuance amount (SUM), and the OLS regression method is used to build model (2).


(2) $$\begin{array}{l} SUM=\partial_{0}+\partial_{1}List+\partial_{2}Post^{*}List+\partial_{3}Size+\partial_{4}Lev+\partial_{5}Tobinq\\ +\partial_{6}Roa+\partial_{7}Cfo+\partial_{8}Growth+\partial_{9}Dual+\partial_{10}Instu+\partial_{11}Big\\ +\partial_{12}Disp+\partial_{13}Soe+\partial_{14}\sum year+\partial_{15}\sum ind+\varepsilon \end{array}$$

For hypothesis 2, the difference in the impact of relaxing short-selling controls on companies’ private placements is tested according to the degree of company equity checks and balances. If the company’s equity balance is included in the regression model for the cross-term test, there may be a serious collinearity problem of three-term cross-product. Therefore, the companies are divided into two groups according to the degree of equity checks and balances, and are tested based on models (1) and (2), respectively. This study expects that the short-selling time point and the short-selling list cross-term (Post^*^List) coefficients is negative in the sample with a higher degree of equity checks and balances.

### Variable Definitions

The definitions of the main variables of the above models are shown in Table 1.

**Table 1:** Variable definitions.

	Variable name	Variable symbol	Variable measure
Explained variables	The company’s tendency to issue additional offerings	SEO	Dummy variable, the value of the additional issuance amount of the company i in the year exceeds the median amount of the additional issuance company in the year, and the value is 1, otherwise it is 0
	The company’s additional issuance amount	SUM	Take the natural logarithm of the additional issuance amount of company i in the year
Explanatory variables	Short selling time	Post	Dummy variable, the value of year t company i is 1 for the short selling target, otherwise it is 0
	Short list	List	Dummy variable, the value of company i is 1 for the short selling target, otherwise it is 0
	Equity balance	Disp	The ratio of the sum of the shareholding ratios of the second to tenth shareholders to the ratio of the first largest shareholder
Control variables	Company Size	Size	The company’s total assets at the end of the year take the natural logarithm
	Assets and liabilities	Lev	The company’s total liabilities at the end of the year to the company’s total assets at the end of the year
	Tobin’s Q	Tobinq	Equity market value plus net debt market value
	Return on total assets	Roa	Net Profit to Total Assets
	Operating income cash content ratio	Cfo	Operating cash flow to operating income
	Growth	Growth	(This year’s operating income – last year’s operating income)/last year’s operating income
	Two jobs in one	Dual	The value of the chairman and the general manager is 1, otherwise it is 0
	Institutional investor shareholding	Instu	Percentage of shares held by institutional investors in company i in year t
	Shareholding ratio of major shareholders	Big	Shareholding ratio of the largest shareholder
	Nature of ownership	Soe	Dummy variable, if the actual controller of the listed company is a state-owned enterprise, the value is 1, otherwise it is 0
	Year	year	Annual dummy variable
	Industry	ind	Industry dummy variable

## Empirical Results Analysis

### Descriptive Statistics

Table 2 reports the descriptive statistical results of the main variables involved in the regression. It can be seen from Table 2 that the average value of the company’s additional issuance tendency (SEO) is 0.069, indicating that the sample that chooses additional issuance accounts for 6.90% of the total sample; the average value of the company’s additional issuance amount (SUM) is 1.131. The average value of the short-selling list (List) is 0.409, that is, the proportion of the short-selling target samples in the total sample is 40.90%; the average value of the short-selling time point (Post) is 0.143, that is, the samples entering the short-selling target account for 14.30% of the total sample. The average value of equity checks and balances (Disp) is 0.176.

**Table 2:** Variable descriptive statistics.

Variable	Number	Mean	Median	Standard deviation	Minimum	Maximum
SEO	8,803	0.069	0	0.254	0	1
SUM	8,803	1.131	0	4.678	0	21.480
List	8,803	0.409	0	0.492	0	1
Post	8,803	0.143	0	0.350	0	1
Disp	8,803	0.176	0.160	0.097	0.003	0.634
Size	8,803	22.020	21.910	1.204	19.060	25.830
Lev	8,803	0.516	0.524	0.204	0.071	1.099
Tobinq	8,803	2.061	1.442	2.096	0.203	15.450
Roa	8,803	0.031	0.028	0.058	−0.220	0.213
Cfo	8,803	0.067	0.066	0.250	−1.372	0.809
Growth	8,803	0.187	0.092	0.640	−0.645	5.510
Dual	8,803	0.161	0	0.367	0	1
Instu	8,803	0.068	0.033	0.101	0	0.575
Big	8,803	0.349	0.333	0.149	0.087	0.758
Soe	8,803	0.543	1	0.498	0	1

### Multiple Regression Results and Analysis

Table 3 reports the test results of hypothesis 1. Columns (1) and (2) take the company’s additional offering selection (SEO) as the dependent variable, and when column (1) does not control the industry, the short-selling time point and the short-selling list cross item (Post^*^List) coefficient is at the 1% significance level. The bottom is negative, indicating that the relaxation of short-selling controls has reduced the company’s options for additional issuance. When column (2) further controls the industry and other variables, the Post^*^List coefficient is also negative at the 1% significance level, which means that the conclusion that relaxing short-selling controls reduces the company’s choice for additional issuance is reliable. Columns (3) and (4) take the company’s additional issuance amount (SUM) as the dependent variable, and when column (3) does not control the industry, the coefficient of the multiplication of the short-selling time point and the short-selling list (Post^*^List) is at the 1% significant level The bottom is negative, indicating that the relaxation of short-selling controls has reduced the company’s additional issuance amount. When further controlling the industry and other variables in column (4), the Post^*^List coefficient is also negative at the 1% significance level, which means that the conclusion that the relaxation of short-selling controls reduces the company’s additional issuance amount is reliable. The test results in Table 3 are in line with the expectations of hypothesis 1. The relaxation of short-selling controls reduces the company’s private placement options and private placement amount.

**Table 3:** Relaxation of short-selling controls and private equity issuance by companies.

Variable	(1)	(2)	(3)	(4)
	SEO	SEO	SUM	SUM
	Probit		OLS	
List	0.062	0.063	−0.190	−0.245^**^
	(1.08)	(1.09)	(−1.64)	(−2.08)
Post^*^List	−0.247^***^	−0.241^***^	−0.783^***^	−0.802^***^
	(−2.89)	(−2.79)	(−3.62)	(−3.68)
Disp	−0.002	−0.013	1.123^***^	1.098^***^
	(−0.05)	(−0.29)	(8.94)	(8.83)
Size	0.003	−0.002	0.490^***^	0.551^***^
	(0.11)	(−0.07)	(7.65)	(8.30)
Lev	0.733^***^	0.720^***^	−2.088^***^	−2.081^***^
	(6.20)	(5.94)	(−8.16)	(−7.95)
Tobinq	0.029^**^	0.031^**^	0.078^**^	0.079^**^
	(2.39)	(2.47)	(2.19)	(2.21)
Roa	−0.984^**^	−0.985^**^	−4.153^***^	−3.764^***^
	(−2.50)	(−2.46)	(−4.78)	(−4.31)
Cfo	0.112	0.094	−0.301^*^	−0.319^*^
	(1.39)	(1.11)	(−1.75)	(−1.79)
Growth	0.144^***^	0.144^***^	0.858^***^	0.867^***^
	(5.67)	(5.63)	(6.43)	(6.58)
Dual	−0.078	−0.077	0.492^***^	0.462^***^
	(−1.33)	(−1.31)	(3.19)	(2.99)
Instu	0.489^***^	0.489^***^	2.778^***^	2.464^***^
	(2.64)	(2.61)	(4.91)	(4.32)
Big	0.043	−0.002	2.489^***^	2.371^***^
	(0.22)	(−0.01)	(5.79)	(5.45)
Soe	−0.085^*^	−0.098^**^	−0.554^***^	−0.543^***^
	(−1.89)	(−2.12)	(−5.32)	(−5.19)
_cons	−2.122^***^	−2.089^***^	−10.608^***^	−11.689^***^
	(−3.86)	(−3.61)	(−7.81)	(−8.12)
year	yes	yes	yes	yes
ind	no	yes	no	yes
*N*	8,803	8,803	8,803	8,803
*R* ^2^	0.038	0.043	0.073	0.081
LR chi2/F	170.480	190.110	17.042	19.700

^*^, ^**^, and ^***^ indicate significant at the significance levels of 10%, 5%, and 1%, respectively (two-tailed test).

Table 4 reports the test results of hypothesis 2. Columns (1) and (2) take the company’s additional issuance selection (SEO) as the dependent variable, and column (1) has a low equity balance, and the coefficients of the short-selling time point and the short-selling list cross-term (Post^*^List) have not passed the significance test, column (2) samples with a high degree of equity balance, the Post^*^List coefficient is negative at the 5% significance level, and columns (1) and (2), the difference between the coefficients between groups has passed the significance test, indicating that the company’s high degree of equity balance is relative to When the degree of equity checks and balances is low, it is more significant to relax the control of short selling and reduce the company’s choice for additional issuance. Columns (3) and (4) take the company’s additional issuance amount (SUM) as the dependent variable, column (3) samples with low equity balance, and the Post^*^List coefficient fails the significance test; column (4) samples with high equity balance, Post^*^ The List coefficient is negative at the 1% significance level, and the difference between the columns (3) and (4) is significant, indicating that when the company’s equity balance is high compared to low, it is more significant to relax the short-selling control and reduce the company’s additional issuance amount. The test in Table 4 confirms the conclusion of hypothesis 2, that is, the company with a high degree of equity checks and balances is more significant than a sample with a low degree of equity checks and balances.

**Table 4:** Deregulation of short selling and private placement of companies: the degree of equity checks and balances.

Variable	(1)	(2)	(3)	(4)
	SEO		SUM	
	High	Low	High	Low
List	−0.164^*^	0.263^***^	−0.588^***^	0.051
	(−1.88)	(3.34)	(−3.00)	(0.39)
Post^*^List	−0.183	−0.291^**^	−1.032	−0.462^**^
	(−1.40)	(−2.47)	(−1.59)	(−2.02)
Size	0.049	−0.059	0.975^***^	0.189^***^
	(1.27)	(−1.53)	(8.39)	(2.70)
Lev	0.648^***^	0.934^***^	−3.149^***^	−0.937^***^
	(4.00)	(5.06)	(−7.57)	(−3.06)
Tobinq	0.040^**^	0.016	0.129^**^	0.045
	(2.48)	(0.77)	(2.31)	(1.09)
Roa	−0.713	−0.905	−4.626^***^	−2.678^**^
	(−1.30)	(−1.49)	(−3.47)	(−2.35)
Cfo	0.070	0.136	−0.584^**^	−0.096
	(0.62)	(1.05)	(−2.02)	(−0.54)
Growth	0.115^***^	0.183^***^	0.840^***^	0.893^***^
	(3.40)	(4.34)	(4.86)	(4.46)
Dual	−0.049	−0.132	0.392^*^	0.415^**^
	(−0.63)	(−1.43)	(1.67)	(2.28)
Instu	0.875^***^	0.295	4.187^***^	1.491^**^
	(3.07)	(1.14)	(4.16)	(2.31)
Big	−0.536^*^	0.139	1.904^**^	1.834^***^
	(−1.67)	(0.59)	(2.03)	(4.75)
Soe	−0.098	−0.127^*^	−0.787^***^	−0.353^***^
	(−1.49)	(−1.93)	(−4.69)	(−3.03)
_cons	−2.880^***^	−1.390	−18.625^***^	−4.206^***^
	(−3.48)	(−1.64)	(−7.41)	(−2.73)
year	yes	yes	yes	yes
ind	yes	yes	yes	yes
*N*	4,400	4,403	4,400	4,403
*R* ^2^	0.058	0.056	0.094	0.050
LR chi2/F	126.160	125.890	12.200	6.04
Difference test value of p	0.037		0.062	

^*^, ^**^, and ^***^ indicate significant at the significance levels of 10%, 5%, and 1%, respectively (two-tailed test).

### Robustness Check

#### Key Metric Replacement

In the previous article, the company’s additional issuance is measured by additional issuance selection and amount. Here, whether additional issuance (SEO-if) and the ratio of additional issuance amount to the company’s market value (Ration) are used as dependent variables to examine the impact of relaxing short-selling controls on the company’s additional issuance. Columns (1) and (2) of Table 5 report the regression results, and the regression coefficient between the short-selling time point and the short-selling list (Short^*^List) is significantly negative, which confirms that the conclusion of hypothesis 1 is robust.

**Table 5:** The robustness test of relaxing short-selling control and the company’s private equity issuance.

Variable	(1)	(2)	(3)	(4)	(5)	(6)
	SEO-if	Ration	SEO	SUM	SEO	SUM
	Key metric replacement		PSM		Short-selling volume	
List	0.242^***^	−0.007^***^	−0.064	−0.162		
	(4.96)	(−2.89)	(−0.39)	(−0.37)		
Post^*^List	−0.332^***^	−0.009^**^	−0.110^*^	−1.226^**^		
	(−4.63)	(−2.44)	(−1.71)	(−2.56)		
SSP					−0.872^**^	−1.253^**^
					(−2.06)	(−2.02)
SSQ					0.022	0.014
					(1.20)	(0.30)
Disp	0.047	0.015^***^	0.012	0.842^***^	−0.306^*^	0.281
	(1.31)	(8.56)	(0.14)	(3.50)	(−1.79)	(0.87)
Size	0.047^**^	0.007^***^	−0.037	0.609^***^	0.037	0.797^***^
	(2.03)	(5.63)	(−0.66)	(3.87)	(0.44)	(4.03)
Lev	0.787^***^	−0.033^***^	0.935^***^	−2.663^***^	1.514^***^	−2.381^**^
	(7.11)	(−6.11)	(2.94)	(−3.04)	(3.25)	(−2.22)
Tobinq	0.046^***^	0.004^***^	0.048^**^	0.212^***^	−0.016	0.316^***^
	(4.39)	(7.91)	(2.06)	(2.99)	(−0.32)	(3.16)
Roa	0.258	−0.016	−2.317^**^	−5.597^**^	0.964	−8.101^**^
	(0.69)	(−0.85)	(−2.45)	(−2.15)	(0.65)	(−2.50)
Cfo	0.041	−0.004	0.283	−0.351	0.226	−0.477
	(0.50)	(−1.04)	(1.44)	(−0.68)	(0.78)	(−0.70)
Growth	0.125^***^	0.021^***^	0.115^**^	0.642^***^	−0.027	0.915^***^
	(5.00)	(14.40)	(2.11)	(3.43)	(−0.19)	(2.62)
Dual	0.014	0.008^***^	−0.001	0.369	−0.011	0.361
	(0.29)	(3.08)	(−0.01)	(1.07)	(−0.07)	(0.90)
Instu	0.507^***^	0.005	0.998^***^	2.428^**^	1.306^**^	4.676^***^
	(2.83)	(0.59)	(2.68)	(2.13)	(2.27)	(3.01)
Big	0.191	0.042^***^	0.644	0.171	0.050	−0.353
	(1.11)	(4.92)	(1.62)	(0.16)	(0.08)	(−0.23)
Soe	−0.226^***^	−0.006^***^	−0.161	−0.854^***^	−0.152	−0.882^***^
	(−5.72)	(−3.19)	(−1.61)	(−3.13)	(−1.15)	(−2.70)
_cons	−2.999^***^	−0.158^***^	−1.018	−11.282^***^	−2.739	−18.157^***^
	(−6.05)	(−6.28)	(−0.83)	(−3.27)	(−1.47)	(−3.18)
year	yes	yes	yes	yes	yes	yes
ind	yes	yes	yes	yes	yes	yes
*N*	8,803	8,803	2,518	2,518	1,259	1,259
*R* ^2^	0.047	0.073	0.071	0.080	0.095	0.061
LR chi2/F	302.410	17.645	77.910	4.799	59.380	2.600

^*^, ^**^, and ^***^ indicate significant at the significance levels of 10%, 5%, and 1%, respectively (two-tailed test).

#### Paired Samples Test

In order to explore the causal relationship between the deregulation of short-selling and the company’s additional issuance, this paper uses the propensity score matching method (PSM) to construct a group of samples from the control group that is closest to the treatment group as the control group. The specific method is as follows: first, use the Logit model to score whether the sample enterprises are inclined to enter the short-selling target; then, use the nearest neighbor matching method to match the samples. Select the target company size, asset-liability ratio, market value, return on total assets and operating income cash content ratio indicators to match. After propensity score matching, 1,383 control group samples were obtained, plus 1,383 experimental group samples, for a total of 2,766 samples. The above matching samples and samples of the experimental group are regressed, and the regression results are reported in columns (3) and (4) of Table 5, and the regression coefficient of the multiplication term (Short^*^List) of the short-selling time point and the short-selling list is obtained, which confirms the conclusion of hypothesis 1 is robust.

#### Detection of Short Selling

China has launched a short-selling mechanism to allow financing transactions for short-selling companies at the same time. Therefore, the reduction of the bond maturity of the target companies of margin financing and securities lending may be caused by the financing mechanism. In order to eliminate this concern, refer to Chu and Fang (2016), taking the target securities lending volume (SSP) as the observation variable, and considering the influence of the financing transaction system and adding the financing volume (SSQ) as the control variable, to detect the selling price. The effect of air volume on the company’s additional issuance, the regression results are reported in the columns (5) and (6) of Table 5. Column (5) takes the company’s additional issuance option (SEO) as the dependent variable, and the coefficient of SSP is negative at the 5% significance level, indicating that the relaxation of short-selling controls reduces the company’s private placement option. Column (6) takes the company’s additional issuance amount (SUM) as the dependent variable, and the coefficient of SSP is negative at the 5% significance level, indicating that the relaxation of short-selling controls reduces the company’s private issuance amount, which confirms that the conclusion of hypothesis 1 is reliable.

## Further Analysis and Discussion

### Financing Constraints

Short-selling controls make it difficult for the market to respond to negative corporate news, leading to market mispricing (Miller, 1977), leading to overvaluation of stock prices in the market, and companies can raise the required funds at a lower cost of capital (Baker et al., 2003), so deregulation of short selling will make the company lose the advantage of low financing cost. According to the financing constraint hypothesis, the source of funds for the company’s investment projects largely depends on external financing, and the relaxation of short-selling controls makes the company’s stock price return to rationality. At this time, the cost of financing by the company through private placement increases, which may reduce the private placement. Therefore, based on the financing constraint hypothesis, relaxing short-selling controls will reduce the company’s private placement.

In order to verify the above conjectures, samples are grouped according to the size of financing constraints, using SA index to calculate the degree of corporate financing constraints, SA = –0.737 × Size + 0.043 × Size2 – 0.04 × Age, where size-company size, age-company age. Taking the median of SA as the benchmark, the group below the median is the group with strong financing constraints (Big), and the group higher than the median is the group with weak financing constraints (Small), respectively, to test the difference of the effect of relaxing short-selling control on the company’s private placement. Table 6 reports the test results. Columns (1) and (2) take the company’s additional offering selection (SEO) as the dependent variable, and the short-selling time point and short-selling list cross item (Post^*^List) coefficients both pass the significance test, but the difference between groups is not significant. Columns (3) and (4) use the company’s additional issuance amount (SUM) as the dependent variable, and the Post^*^List coefficients have also passed the significance test, and the differences between groups are not significant. The test in Table 6 confirms that the size of financing constraints does not affect the effect of relaxing short-selling controls on the company’s private placement, excluding the effect of relaxing short-selling controls reducing the company’s private placement through the financing constraint mechanism.

**Table 6:** Deregulation of short selling and private placement of companies: the size of financing constraints.

Variable	(1)	(2)	(3)	(4)
	SEO		SUM	
	Small	Big	Small	Big
List	0.122	0.021	−0.281	−0.393^***^
	(1.56)	(0.23)	(−1.51)	(−2.75)
Post^*^List	−0.216^*^	−0.337^**^	−0.805^***^	−0.630^*^
	(−1.92)	(−2.01)	(−2.68)	(−1.78)
Disp	−0.072	0.047	1.163^***^	1.055^***^
	(−1.11)	(0.78)	(5.96)	(6.53)
Size	−0.036	0.020	0.342^***^	1.138^***^
	(−0.77)	(0.33)	(2.88)	(8.55)
Lev	0.890^***^	0.605^***^	−2.992^***^	−1.576^***^
	(3.70)	(4.19)	(−5.10)	(−5.67)
Tobinq	0.009	0.042^***^	0.282^***^	0.148^***^
	(0.29)	(2.61)	(2.73)	(3.34)
Roa	−0.257	−1.481^***^	−9.042^***^	−3.382^***^
	(−0.35)	(−2.96)	(−4.46)	(−3.54)
Cfo	0.045	0.203	−0.554^*^	−0.225
	(0.37)	(1.57)	(−1.70)	(−1.27)
Growth	0.144^***^	0.163^***^	1.029^***^	0.708^***^
	(4.03)	(4.19)	(5.15)	(4.18)
Dual	−0.154^*^	−0.033	0.539^**^	0.367^**^
	(−1.67)	(−0.42)	(2.00)	(2.03)
Instu	0.510^**^	0.288	2.531^***^	1.990^**^
	(2.21)	(0.83)	(3.21)	(2.51)
Big	−0.273	0.375	2.118^***^	2.819^***^
	(−0.97)	(1.24)	(3.22)	(4.88)
Soe	−0.031	−0.164^**^	−0.398^**^	−0.690^***^
	(−0.46)	(−2.46)	(−2.35)	(−5.26)
_cons	−1.367	−2.592^**^	−7.047^***^	−24.421^***^
	(−1.34)	(−2.00)	(−2.67)	(−8.52)
year	yes	yes	yes	yes
ind	yes	yes	yes	yes
*N*	4,405	4,398	4,405	4,398
*R* ^2^	0.042	0.060	0.084	0.097
LR chi2/F	95.140	130.490	10.830	12.000
*p*-value	0.218		0.149	

^*^, ^**^, and ^***^ indicate significant at the significance levels of 10%, 5%, and 1%, respectively (two-tailed test).

### Whether to Combine the Two Roles

The short-selling mechanism motivates outside investors to profit by collecting bad news about the company. Company managers learn private information from company fundamentals reflected in stock prices and incorporate this information into investment decisions (Chen et al., 2007). Then the short-selling behavior of short-selling investors outside the company will put pressure on the company’s managers to actively change their financing decisions and improve the quality of earnings. Therefore, based on the corporate governance hypothesis, the short-selling mechanism may prompt companies to reduce the encroachment of private placements.

In order to verify the above conjectures, here we examine the differences in the effect of relaxing short-selling controls on companies’ private placements according to whether the chairman and the general manager are dual. Table 7 reports the test results. Columns (1) and (2) take the company’s additional offering selection (SEO) as the dependent variable, and column (1) when Dual = 1, that is, when the two positions are combined, the short-selling time point and the short-selling list multiplication item (Post^*^List) coefficient fails to pass Significance test; column (2) when Dual = 0, i.e., non-combination of two positions, the Post^*^List coefficient is negative at the 1% significance level, indicating that the relaxation of short-selling controls has reduced the company’s choice for private placement; the difference is significant, indicating that when the job is not in the same position, the relaxation of short-selling controls and the reduction of additional issuance by the company are more significant. Columns (3) and (4) take the company’s additional issuance amount (SUM) as the dependent variable, and the Post^*^List coefficients have passed the significance test, and there is no significant difference between the groups. The test in Table 7 shows that when the corporate governance is good, the relaxation of short-selling controls reduces the company’s private placement options, but does not affect the company’s private placement amount, excluding the mechanism of relaxing short-selling controls to reduce the company’s private placement through corporate governance.

**Table 7:** Deregulation of short selling and private placement of companies: whether to combine the two roles.

Variable	(1)	(2)	(3)	(4)
	SEO		SUM	
	Dual = 1	Dual = 0	Dual = 1	Dual = 0
List	−0.264	0.101	0.563	−0.373^***^
	(−1.48)	(1.63)	(1.49)	(−3.07)
Post^*^List	−0.058	−0.249^***^	−2.026^***^	−0.535^**^
	(−0.25)	(−2.66)	(−3.27)	(−2.32)
Disp	0.164	−0.035	1.815^***^	0.943^***^
	(1.59)	(−0.70)	(5.49)	(7.08)
Size	−0.072	0.004	0.482^**^	0.548^***^
	(−1.00)	(0.14)	(2.40)	(7.85)
Lev	0.978^***^	0.666^***^	−1.003	−2.298^***^
	(3.16)	(5.01)	(−1.47)	(−8.12)
Tobinq	0.016	0.032^**^	0.087	0.080^**^
	(0.62)	(2.26)	(0.98)	(2.03)
Roa	0.812	−1.310^***^	−3.017	−4.179^***^
	(1.01)	(−2.85)	(−1.38)	(−4.35)
Cfo	−0.016	0.116	−0.615	−0.208
	(−0.08)	(1.23)	(−1.27)	(−1.12)
Growth	0.004	0.166^***^	1.104^***^	0.766^***^
	(0.06)	(5.82)	(3.04)	(5.58)
Instu	0.742	0.447^**^	1.912	2.653^***^
	(1.34)	(2.24)	(1.14)	(4.39)
Big	0.345	−0.035	6.351^***^	1.583^***^
	(0.59)	(−0.16)	(4.37)	(3.58)
Soe	−0.111	−0.098^**^	−0.539^*^	−0.530^***^
	(−0.81)	(−1.99)	(−1.74)	(−4.74)
_cons	−0.430	−2.336^***^	−12.054^***^	−11.242^***^
	(−0.28)	(−3.65)	(−2.64)	(−7.48)
year	yes	yes	yes	yes
ind	yes	yes	yes	yes
*N*	1,417	7,386	1,417	7,386
*R* ^2^	0.075	0.046	0.157	0.069
LR chi2/F	50.240	171.570	7.110	14.240
*p*-value	0.000		0.143	

^*^, ^**^, and ^***^ indicate significant at the significance levels of 10%, 5%, and 1%, respectively (two-tailed test).

### Information Environment

The relaxation of short-selling control provides short-selling investors with a way to tap the company’s “bad news” investment profits, and more “bad news” for short-selling target companies enter the market, making the information environment of short-selling target companies and non-short-selling target companies have the same information environment. If there is a large difference, then we expect that the relaxation of short-selling control will affect the company’s private placement behavior through the information environment mechanism. Information transparency is an important basis for investors to invest in asset portfolios based on the information disclosed by companies, and it is also an important indicator to measure the effective operation of the capital market. The higher the information transparency is, the lower the information asymmetry between the company’s internal major shareholders and management and external investors and other stakeholders is and the lower the possibility of investors being deceived is, otherwise the higher the possibility of investors being deceived is. After the short-selling control is relaxed, the major shareholders of companies with better information environment are less likely to transmit benefits through private placement, and the risk of being short-sold is relatively lower for major shareholders. Therefore, the impact of relaxing short-selling controls on the company’s private placement relatively weaker.

In order to verify the above conjectures, based on the median of each year’s information transparency (Opacity) of listed companies as the benchmark, they are divided into two groups, high and low information transparency, to test the difference in the impact of relaxing short-selling controls on companies’ private placements. Table 8 reports regression results. Information transparency is measured by the sum of the controllable accruals in the 3 years before the company i year t. The larger the value, the lower the information transparency, and the smaller the value, the higher the information transparency. Columns (1) and (2) take the company’s additional offering selection (SEO) as the dependent variable, and column (1) when the company’s information transparency is low, the short-selling time point and the short-selling list cross item (Post^*^List) coefficient is significant at 5%. It is negative below the level, indicating that the relaxation of short-selling control reduces the company’s choice for additional issuance; column (2) when the company’s information transparency is high, the Post^*^List coefficient fails the significance test, and the difference between groups is significant, indicating that when the company’s information environment is low, relaxing Short-selling controls reduces the company’s additional issuance more significantly. Columns (3) and (4) use the company’s additional issuance amount (SUM) as the dependent variable, and also get the same results as columns (1) and (2). The test in Table 8 confirms that when the company’s information environment is poor, compared with when the company’s information environment is good, it is more significant to relax short-selling controls and reduce the company’s private placement.

**Table 8:** Deregulation of short selling and private placement of companies: the level of information transparency.

Variable	(1)	(2)	(3)	(4)
	SEO		SUM	
	Low	High	Low	High
List	0.184^**^	−0.039	−0.264	−0.271
	(2.22)	(−0.47)	(−1.52)	(−1.63)
Post^*^List	−0.296^**^	−0.195	−1.236^***^	−0.300
	(−2.45)	(−1.54)	(−4.23)	(−0.91)
Disp	0.015	−0.044	1.291^***^	0.941^***^
	(0.22)	(−0.75)	(6.91)	(5.65)
Size	−0.007	0.001	0.615^***^	0.527^***^
	(−0.17)	(0.04)	(6.68)	(5.34)
Lev	0.968^***^	0.501^***^	−1.688^***^	−2.785^***^
	(6.10)	(2.62)	(−5.00)	(−6.54)
Tobinq	0.014	0.051^***^	0.109^**^	0.023
	(0.83)	(2.73)	(2.28)	(0.41)
Roa	−0.521	−1.576^**^	−3.418^***^	−4.542^***^
	(−1.02)	(−2.46)	(−2.99)	(−3.29)
Cfo	0.040	0.165	−0.164	−0.504
	(0.39)	(1.05)	(−0.78)	(−1.49)
Growth	0.150^***^	0.146^***^	0.609^***^	1.281^***^
	(4.46)	(3.61)	(3.82)	(5.47)
Dual	−0.029	−0.138	0.573^***^	0.272
	(−0.37)	(−1.48)	(2.70)	(1.23)
Instu	0.270	0.692^***^	2.548^***^	2.256^***^
	(0.96)	(2.72)	(3.03)	(2.90)
Big	0.221	−0.191	2.819^***^	1.838^***^
	(0.77)	(−0.65)	(4.73)	(2.86)
Soe	−0.217^***^	0.009	−0.609^***^	−0.461^***^
	(−3.39)	(0.14)	(−4.09)	(−3.12)
_cons	−2.538^***^	−1.726^**^	−13.502^***^	−10.482^***^
	(−3.04)	(−2.07)	(−6.73)	(−4.93)
year	yes	yes	yes	yes
ind	yes	yes	yes	yes
*N*	4,403	4,400	4,403	4,400
*R* ^2^	0.062	0.047	0.091	0.081
LR chi2/F	137.840	103.120	11.510	9.870
*p*-value	0.039		0.000	

^*^, ^**^, and ^***^ indicate significant at the significance levels of 10%, 5%, and 1%, respectively (two-tailed test).

In addition to the company’s internal information environment, the number of analysts tracking the company is an important indicator of the company’s external information environment. Here, it is also divided into two groups based on the median number of analysts tracking listed companies (Analyst) to test the difference in the impact of relaxation of short-selling controls on companies’ private placements. Table 9 reports the regression results. Columns (1) and (2) take the company’s additional offering selection (SEO) as the dependent variable, and column (1) when the number of analysts following is small, the coefficient of the multiplication factor (Post^*^List) between the short-selling time point and the short-selling list (Post^*^List) is negative at 5% significance level, indicating that the relaxation of short-selling control has reduced the company’s choice for additional issuance; Column (2) when the number of analysts following is large, the Post^*^List coefficient does not pass the significance test, and the difference between groups is significant, indicating that when it is less, the relaxation of short-selling controls to reduce the company’s additional issuance is more significant. Columns (3) and (4) use the company’s additional issuance amount (SUM) as the dependent variable, and also get the same results as columns (1) and (2). The test in Table 9 confirms that the relaxation of short-selling control reduces the company’s additional issuance more significantly when the number of analysts following is less than when the number of analysts is more.

**Table 9:** Deregulation of short selling and private placements of companies: analysts track numbers.

Variable	(1)	(2)	(3)	(4)
	SEO		SUM	
	Small	Big	Small	Big
List	0.091	−0.061	−0.346^*^	−0.277
	(0.94)	(−0.64)	(−1.77)	(−1.12)
Post^*^List	−0.448^***^	−0.055	−1.115^***^	−0.583
	(−2.67)	(−0.39)	(−2.78)	(−1.53)
Disp	−0.017	−0.036	1.357^***^	0.945^***^
	(−0.25)	(−0.37)	(6.55)	(3.44)
Size	−0.072	−0.107^*^	0.723^***^	0.461^***^
	(−1.38)	(−1.92)	(5.39)	(3.33)
Lev	0.967^***^	0.682^**^	−1.982^***^	−5.497^***^
	(4.27)	(2.20)	(−3.77)	(−6.34)
Tobinq	0.059^***^	0.020	0.150^*^	0.175^**^
	(2.94)	(0.75)	(1.83)	(2.23)
Roa	−2.124^***^	−2.456^**^	−1.781	−18.643^***^
	(−3.07)	(−2.38)	(−0.98)	(−6.75)
Cfo	0.169	0.109	−0.732^**^	−0.612
	(1.12)	(0.53)	(−2.08)	(−1.00)
Growth	0.179^***^	0.143^**^	1.053^***^	1.428^***^
	(4.09)	(2.57)	(4.64)	(4.07)
Dual	−0.120	−0.120	0.526^**^	0.507
	(−1.21)	(−1.09)	(2.02)	(1.50)
Instu	−0.097	0.530^*^	1.485^*^	3.823^***^
	(−0.29)	(1.81)	(1.73)	(3.60)
Big	−0.331	0.217	3.451^***^	1.417
	(−0.97)	(0.54)	(4.62)	(1.40)
Soe	−0.005	−0.017	−0.526^***^	−0.686^***^
	(−0.07)	(−0.21)	(−2.94)	(−3.16)
_cons	−0.743	0.477	−15.795^***^	−7.453^**^
	(−0.69)	(0.40)	(−5.40)	(−2.42)
year	yes	yes	yes	yes
ind	yes	yes	yes	yes
*N*	4,405	4,398	4,405	4,398
*R* ^2^	0.075	0.061	0.103	0.092
LR chi2/F	124.190	91.600	9.290	7.860
p-value	0.000		0.027	

^*^, ^**^, and ^***^ indicate significant at the significance levels of 10%, 5%, and 1%, respectively (two-tailed test).

## Research Conclusion and Implications

Based on the quasi-natural experiment of China’s gradual relaxation of short-selling control in 2010, this paper examines the impact of the relaxation of short-selling control on the company’s private placement. Taking China’s A-share listed companies in Shanghai and Shenzhen stock markets from 2007 to 2015 as a sample, a double-difference model is constructed to test that the relaxation of short-selling control reduces the company’s private placement tendency and amount, and this effect is mainly reflected in the sample with a high degree of equity checks and balances. Use other companies’ private placement alternative indicators, propensity score matching (PSM) and short-selling volume to test to verify that the above conclusions are robust. Further analysis finds that when the size of the company’s financing constraints is different, there is no difference in the impact of relaxing short-selling controls on the company’s private placement options and amount; it has no effect on the company’s private placement amount, indicating that the relaxation of short-selling controls is not through financing constraints and corporate governance mechanisms to inhibit the company’s private placement. Significantly, it shows that the relaxation of short-selling control policy and supervision inhibits the company’s private placement through the information environment mechanism, and promotes the sustainable development of enterprises. It not only improves the literature on the external governance role of the policy supervision of the short-selling mechanism, but also enriches the governance research of the company’s private placement of benefits, and promotes the sustainable development of enterprises.

The conclusion of the paper confirms that the short-selling mechanism has external corporate governance effects in China. Therefore, with the development and improvement of China’s securities market, the gradual relaxation of the short-selling control capital market policy and supervision system will not only help improve the efficiency of stock market pricing, but also prompt companies to improve the quality of accounting information, improve investor protection, and promote corporate sustainability. The conclusions of this paper further enrich the research on the impact of short-selling mechanisms on corporate behavior, and provide evidence of the positive impact of China’s emerging market countries. However, due to the short time since China launched the short-selling mechanism and the complex information environment of Chinese listed companies, the research on the impact of the short-selling mechanism on corporate behavior needs to provide further empirical evidence in more dimensions to enrich people’s understanding of the short-selling mechanism in China’s securities market. The understanding of economic consequences provides strong theoretical support for policy formulation and regulatory authorities.

## Author’s Note

Panna Wang (1980), associate professor, Ph.D., School of Accounting, Chongqing University of Technology, research direction is capital market finance and accounting. Chun Su (1985), lecturer at the School of Accounting, Chongqing University of Technology, with a research direction of corporate governance. Botao Xu (1978), associate professor at the School of Accounting, Chongqing University of Technology, with research interests in finance and accounting.

## Data Availability Statement

The original contributions presented in the study are included in the article/supplementary materials, further inquiries can be directed to the corresponding author.

## Author Contributions

All authors listed have made a substantial, direct, and intellectual contribution to the work and approved it for publication.

## Funding

This study received funding from the following programmes: “Research on the Influence Mechanism of Capital Market Opening on Auditors’ Decision-Making” (2020BS51), “Research on the governance effect and policy optimization of VAT reduction on the manufacturing industry’s departure from the real to the virtual” (22SKJD115), “Research on the Influence and Economic Consequences of Capital Market Opening on Information Intermediaries” (2019ZD25), and “Excess Control of Family Board Seats, Investment Decisions and Enterprise Value” (2021ZDR023).

## Conflict of Interest

The authors declare that the research was conducted in the absence of any commercial or financial relationships that could be construed as a potential conflict of interest.

## Publisher’s Note

All claims expressed in this article are solely those of the authors and do not necessarily represent those of their affiliated organizations, or those of the publisher, the editors and the reviewers. Any product that may be evaluated in this article, or claim that may be made by its manufacturer, is not guaranteed or endorsed by the publisher.
